# Supplementary figures and images for: Cadherin 11 Inhibition Downregulates β-catenin, Deactivates the Canonical WNT Signalling Pathway and Suppresses the Cancer Stem Cell-Like Phenotype of Triple Negative Breast Cancer
Source: J Clin Med. 2019 Jan 27;8(2):148. doi: 10.3390/jcm8020148 (PMC6407101; doi:10.3390/jcm8020148)

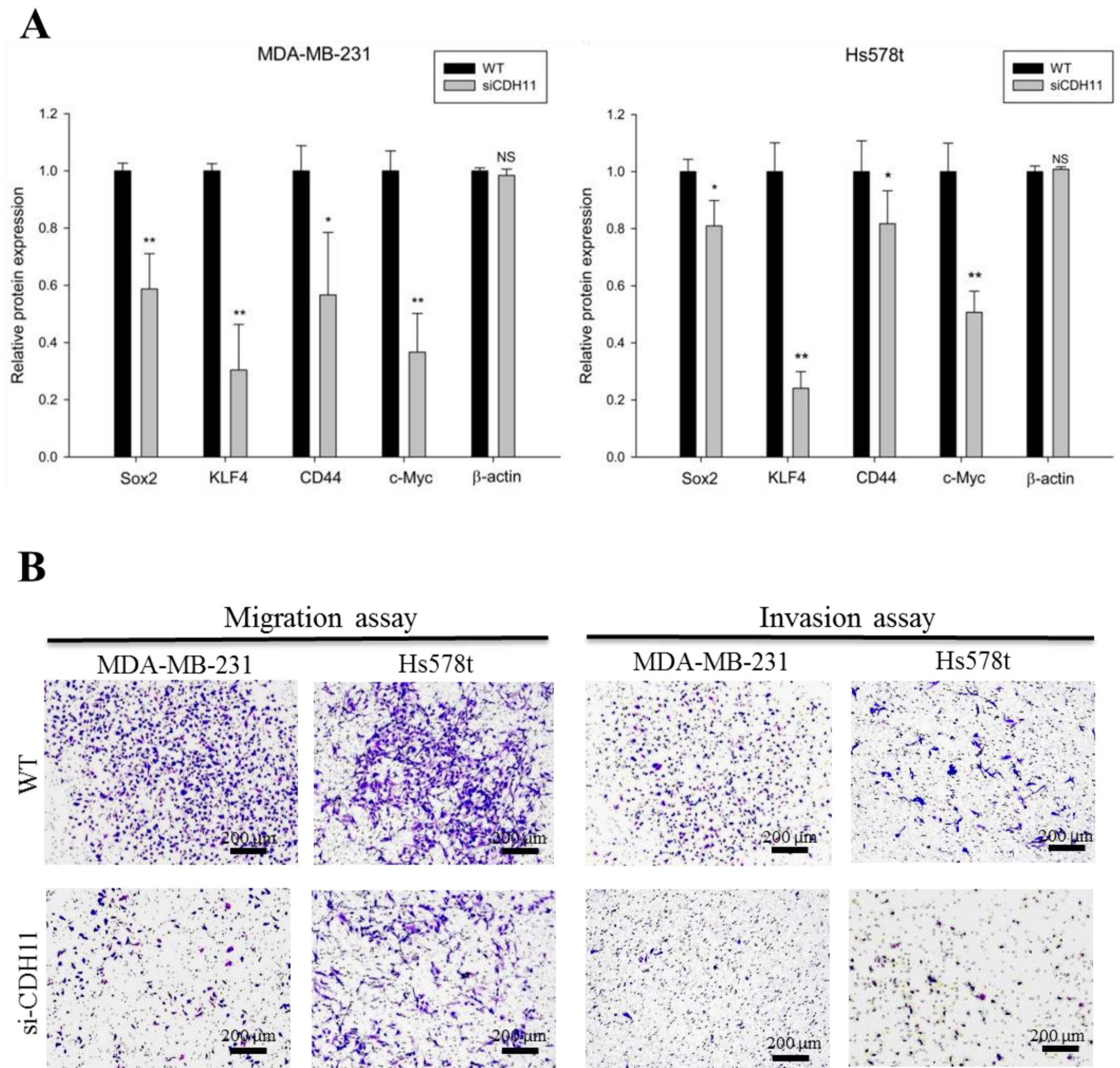

Supplementary Figure 1

Primary culture from  
tumor xenografted mice

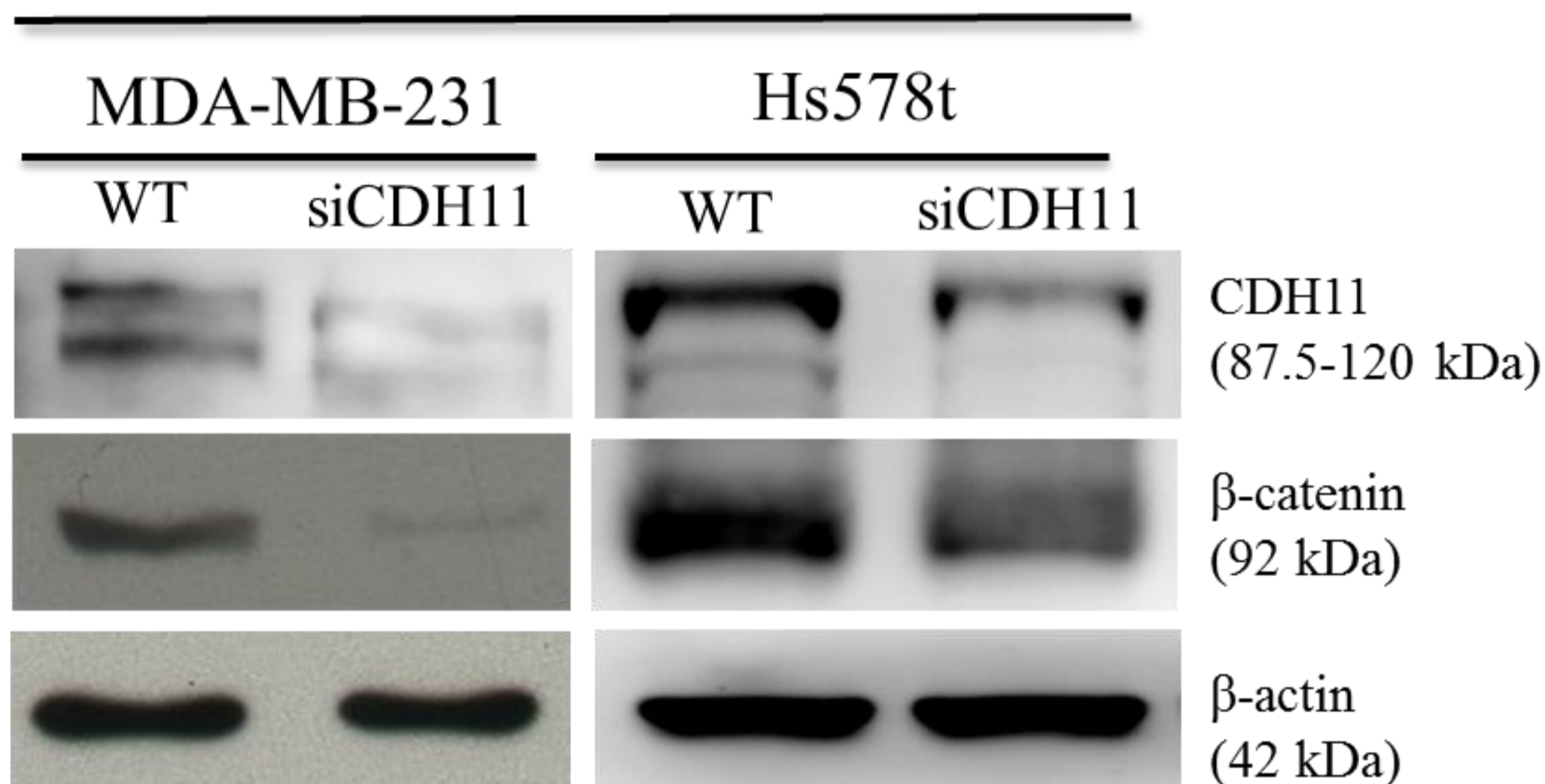

Supplementary Figure 2

Supplement: Supplementary file 1 [file jcm-08-00148-s001.pdf]
